# Prevenção de tromboembolismo venoso em hospital com perfil oncológico: como melhorá-la?

**DOI:** 10.1590/1677-5449.003216

**Published:** 2016

**Authors:** Fabiano Luiz Erzinger, Marcela Bechara Carneiro

**Affiliations:** 1 Hospital Erasto Gaertner, Serviço de Cirurgia Vascular, Curitiba, PR, Brasil.; 2 Hospital Erasto Gaertner, Setor de Farmácia Hospitalar, Curitiba, PR, Brasil.

**Keywords:** câncer, tromboembolismo, prevenção

## Abstract

**Contexto:**

Complicações do tromboembolismo venoso são encontradas frequentemente em pacientes internados, tanto em condições clínicas quanto em pós-operatórios.

**Objetivo:**

Verificar a quimioprofilaxia utilizada para tromboembolismo venoso em pacientes oncológicos internados, antes e após a realização de um programa de esclarecimento da sua importância.

**Métodos:**

Estudo de corte transversal realizado em três momentos distintos: inicialmente antes do programa de conscientização da importância da profilaxia do tromboembolismo venoso, durante o período em que foi realizada e um ano após a etapa anterior. Para fins estatísticos, os pacientes foram divididos em alto risco e baixo risco, e estratificados quanto a erro na quimioprofilaxia em: precisavam, mas não fizeram profilaxia; não precisavam, mas fizeram profilaxia; fizeram profilaxia não padronizada; e não podiam, mas fizeram profilaxia.

**Resultados:**

Foram avaliados 399 pacientes internados, sendo 56 pacientes antes do início do programa de conscientização, 255 durante o programa e 88 após um ano. Antes da realização da semana de conscientização, apenas 35,7% dos pacientes estavam recebendo a quimioprofilaxia adequada; após a semana de conscientização, houve um aumento do número de prescrições corretas, que passou para 63,9% (p < 0,001). Após um ano sem as aulas de conscientização, a manutenção da quimioprofilaxia não foi tão eficaz, com uma tendência ao aumento do número de profilaxias incorretas (p = 0,081).

**Conclusão:**

A quimioprofilaxia é utilizada em uma porcentagem muito pequena nos pacientes internados, sendo necessários programas de esclarecimento de sua importância na prevenção do tromboembolismo venoso e a realização de monitoramento contínuo para auxiliar na sua prescrição.

## INTRODUÇÃO

A principal causa evitável de óbito hospitalar é o tromboembolismo pulmonar (TEP), que na maioria das vezes é consequência de trombose venosa profunda (TVP). Esse conjunto é denominado tromboembolismo venoso (TEV), complicação comum durante e após a hospitalização por doença médica aguda ou cirurgia^1^^-^ 3.

Além do risco agudo de mortalidade, o TEV está associado ao risco de desenvolvimento em longo prazo de síndrome pós-trombótica e hipertensão pulmonar crônica. Essas complicações contribuem substancialmente para morbidade, afastamento das atividades laborais e gestão de custos em saúde^4^.

Aproximadamente 70% a 80% das embolias pulmonares (EPs) diagnosticadas *post mortem* não têm suspeita clínica nem diagnóstico prévio devido à sua causa e por suas complicações serem frequentemente silenciosas. Em função dessas características, deve-se estabelecer a profilaxia como medida segura e eficaz nos pacientes que apresentam fatores de riscos para a sua ocorrência^5^.

A doença oncológica isolada é um fator de risco importante para a ocorrência de TEV. Caso o paciente seja submetido a um procedimento cirúrgico, o risco aumenta em até duas vezes para TVP e três vezes para TEP na comparação com pacientes não oncológicos^6^, o que demonstra o maior cuidado que se deve ter na atenção da aplicação de medidas preventivas nesse perfil de paciente.

A realidade da profilaxia do TEV nos hospitais brasileiros vem sendo estudada nas duas últimas décadas. As constatações são de que a maioria dos pacientes internados, clínicos e/ou cirúrgicos, não recebem as medidas profiláticas adequadas, apesar de apresentarem fatores de risco para o seu desenvolvimento^7^^-^ 11. No entanto, existem poucos estudos com o objetivo de formular estratégias e orientação na forma de programas de tromboprofilaxia em nível hospitalar^11^^,^ 12.

Para melhorar a frequência e a qualidade da utilização de profilaxia do TEV, Machado^13^ recomenda a formação de uma equipe multidisciplinar, que deve entender a importância da quimioprofilaxia^14^^,^ 15. O estudo de Rocha et al.^11^ propõe a criação de uma comissão em cada hospital para incentivar a profilaxia do TEV através de palestras educativas.

O objetivo deste estudo foi avaliar a qualidade da quimioprofilaxia para TEV em pacientes clínicos ou cirúrgicos internados em um hospital oncológico, antes e após a realização de um programa continuado de esclarecimento (PCE) da importância e necessidade desses procedimentos.

## MÉTODO

Foi realizado no Hospital Erasto Gaertner, hospital-escola com perfil oncológico, um estudo de corte transversal durante um único dia, em três momentos distintos: inicialmente antes do PCE da importância da profilaxia do TEV em pacientes internados, durante o período em que foi realizada a conscientização e um ano após a realização do PCE.

O comitê institucional local de ética em pesquisa concedeu aprovação (CAAE 05040012.9.0000.0098) para a realização de todas as fases do estudo e do termo de consentimento livre e esclarecido (TCLE) assinado por todos os pacientes estudados.

O programa de conscientização ocorreu em 2012, 2013 e 2014, e era constituído de uma semana de palestras padronizadas de educação continuada, ministradas por especialistas na área de TEV, para médicos do corpo clínico, médicos residentes, enfermeiros, fisioterapeutas e integrantes da farmácia clínica. Continha informações sobre dados epidemiológicos da profilaxia em pacientes clínicos ou cirúrgicos, abordagem das diretrizes nacionais de profilaxia em TEV, importância da avaliação diária do paciente e cuidados na administração das medicações profiláticas. Para auxiliar na sedimentação da importância da prescrição e na decisão da profilaxia, além das palestras, houve também discussão de casos clínicos e distribuição de materiais educativos sobre o algoritmo da profilaxia existente no prontuário eletrônico vigente no hospital, que é diferente para pacientes internados por condições clínicas ou por condições cirúrgicas.

As coletas de informações sobre o risco de TEV e a quimioprofilaxia utilizada foram realizadas conforme os protocolos para pacientes clínicos e para pacientes cirúrgicos previamente publicados em suplemento deste jornal^16^^-^ 19, através de perguntas diretas ao paciente ou ao seu representante legal, e consulta ao seu prontuário eletrônico, com o intuito de melhorar a qualidade das informações sobre as condições do paciente e suas comorbidades. Foram incluídos os pacientes que estavam internados no dia da coleta dos dados e que aceitaram participar do estudo (ou cujo representante legal aceitou), após terem lido, recebido explicações e esclarecido dúvidas com relação ao TCLE. Os critérios de exclusão foram: pacientes menores de 18 anos, gestantes, pacientes em tratamento para TEV, pacientes que iriam realizar cirurgia naquele dia, pacientes que estavam de alta e pacientes que não se conseguiu obter todas as informações para o preenchimento do protocolo.

Foi realizado um estudo antes das palestras informativas de profilaxia para TEV e entre um e três meses após a semana do PCE, nos anos de 2012, 2013 e 2014. Já no ano de 2015, não foi realizada nenhuma forma de conscientização sobre TEV, e a coleta de dados foi realizada um ano após a coleta de 2014. Durante o período do estudo, os médicos não foram avisados previamente sobre quais os dias em que iriam ocorrer as entrevistas com os pacientes. Para fins estatísticos, os pacientes foram divididos em alto risco ou baixo risco para a ocorrência do TEV. Os pacientes de alto risco eram aqueles que necessitavam de profilaxia ou que apresentavam risco moderado e também necessitariam dela, e os de baixo risco eram aqueles que não necessitavam de profilaxia. Os pacientes que receberam a quimioprofilaxia errada foram ainda estratificados em quatro situações possíveis: precisavam, mas não fizeram profilaxia; não precisavam, mas fizeram profilaxia; fizeram profilaxia não padronizada; e não podiam, mas fizeram profilaxia.

Foi realizada uma análise estatística para avaliação da associação entre as classificações de profilaxia nas três avaliações (antes, após as aulas de conscientização e após um ano sem conscientização). Foi considerado o teste qui-quadrado com valores de p < 0,05 indicando significância estatística. Os dados foram analisados com o programa computacional IBM SPSS Statistics v.20.0®.

## RESULTADOS

No período de 2012 a 2015, foram avaliados 399 pacientes internados no Hospital Erasto Gaertner, sendo 226 pacientes clínicos e 173 pacientes cirúrgicos (Tabela 1), e a maioria era de alto risco para o desenvolvimento de TEV (66,41%). O grupo de pacientes cirúrgicos foi o que apresentou a maior percentagem (75,14%) de alto risco.

**Tabela 1:** Relação dos pacientes internados e o risco de TEV.

**Avaliação**	**Risco/paciente**	**Tratamento**		
		**Clínico**	**Cirúrgico**	**Geral**
Antes das aulas de conscientização (maio/2012)	Alto	19 (59,4%)	22 (91,7%)	41 (73,2%)
	Baixo	13 (40,6%)	2 (8,3%)	15 (26,8%)
	Total	32	24	56
Após as aulas de conscientização	Alto	86 (65,6%)	80 (64,5%)	166 (65,1%)
	Baixo	45 (34,4%)	44 (35,5%)	89 (34,9%)
	Total	131	124	255
Após um ano sem conscientização (2015)	Alto	30 (47,6%)	23 (92%)	53 (60,2%)
	Baixo	33 (52,4%)	2 (8%)	35 (39,8%)
	Total	63	25	88

O fator de risco para TEV mais frequentemente encontrado foi a presença de doença oncológica (87,78%); nos pacientes internados por razões clínicas, essa ocorrência foi de 96,31%, e nos pacientes cirúrgicos, de 78,37%. Nos pacientes clínicos, ainda foram encontrados, de maneira mais frequente, os seguintes fatores de risco: imobilização por tempo prolongado (64,41%), idade maior que 55 anos (57,66%) e quadro de infecção (34,35%). Contraindicações à quimioprofilaxia ocorreram em 90 pacientes (22,55%), sendo as principais plaquetopenia abaixo de 50.000/mm^3^ (45,71%) e sangramento ativo (42,85%); esses fatores ocorreram em aproximadamente 1/4 (26,41%) dos pacientes que apresentavam alto risco para o desenvolvimento de TEV.

Dos 399 pacientes internados nesse período, foram avaliados 56 pacientes antes do início do PCE, 255 pacientes durante o programa e 88 pacientes após um ano do último programa.

De maneira geral, a quimioprofilaxia correta foi prescrita para 213 pacientes (53,38%). Dos 46,62% que receberam quimioprofilaxia errada, a grande maioria – 58 pacientes (52,68%) – eram de alto risco para TEV e ficaram sem receber quimioprofilaxia. Foi utilizada a heparina de baixo peso molecular (HBPM) em 87,2% dos pacientes, isso porque a diferença de custos em relação à heparina não fracionada (HNF) era mínima, chegando a ser menor que 3% ao dia, sem contar a diferença de custos indiretos e posologia.

Antes da realização da semana de conscientização, apenas 35,7% dos pacientes estavam recebendo a quimioprofilaxia adequada. Já dos 36 pacientes que a receberam de forma errada, mais da metade (55,6%) eram pacientes de alto risco e ficaram sem recebê-la.

**Figura 1:**
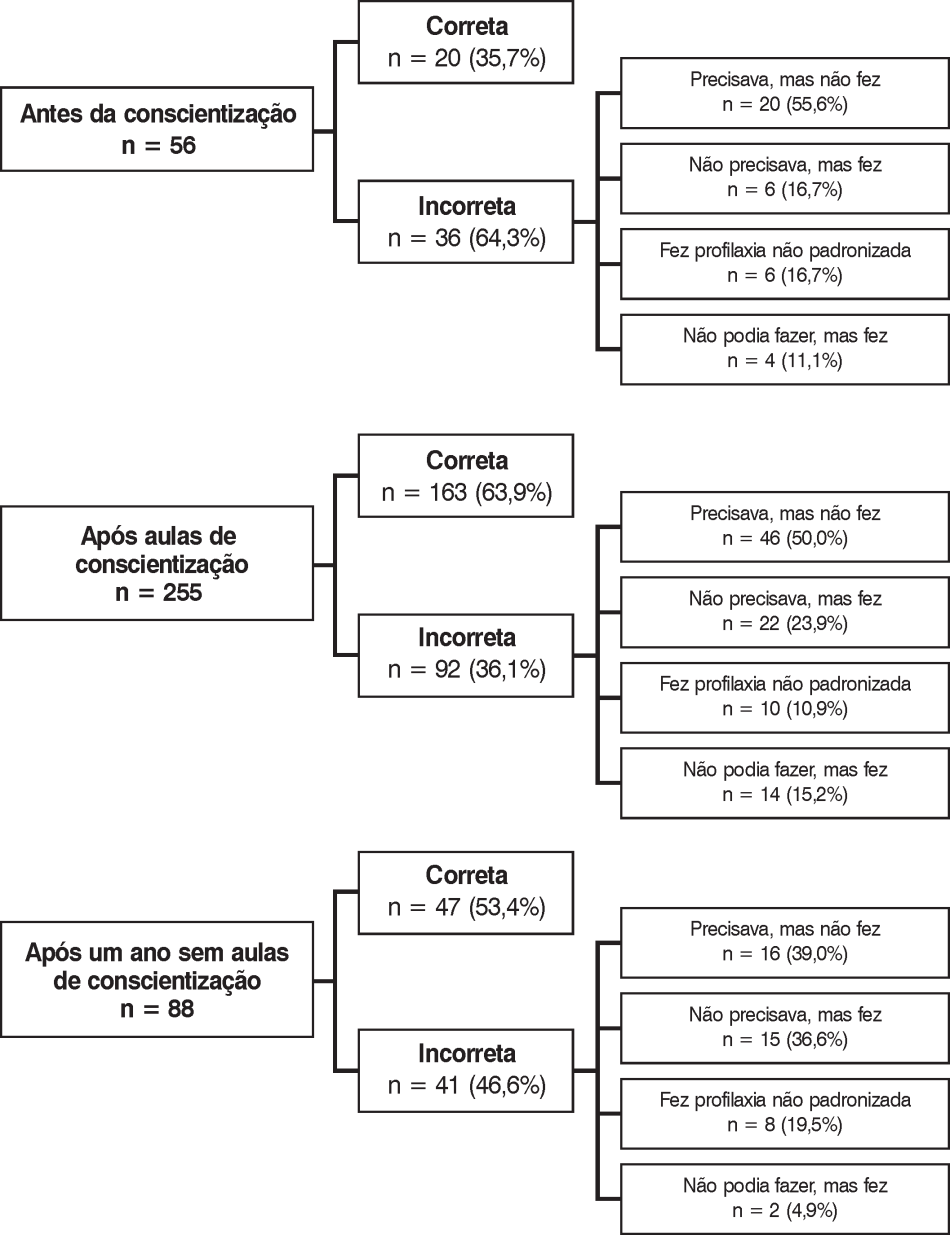
Performance da quimioprofilaxia ao longo do estudo.

Após a semana de conscientização, houve um aumento estatisticamente significativo do número de prescrições corretas de quimioprofilaxia, que passaram de 35,7% para 63,9% (p < 0,001), o que demonstra a efetividade do PCE instituído no hospital. Porém, ainda foi insuficiente, pois cerca de 1/3 (186) dos pacientes internados a receberam de forma errada. Por outro lado, de uma maneira não significativa, houve uma tendência de prescrever mais quimioprofilaxia, que ocorreu em 23,9% dos pacientes que não a necessitariam por serem de baixo risco, o que demonstra uma preocupação em avaliar a possibilidade de proteção contra TEV nos pacientes internados (Figura 1).

Ocorreu também, mesmo após um ano sem as aulas de conscientização (Tabela 2), a manutenção da melhora da prescrição da quimioprofilaxia. Não foi tão eficaz quando comparada ao período em que se realizava a semana de palestras, havendo uma tendência ao aumento das profilaxias incorretas (p = 0,081), mas ainda com menor número de profilaxias incorretas do que antes das aulas (p < 0,001).

**Tabela 2:** Persistência da profilaxia correta após a conscientização.

**Profilaxia**	**Antes das aulas de conscientização (maio/2012)**		**Após as aulas de conscientização**		**Após um ano sem conscientização (2015)**	
	**n**	**%**	**n**	**%**	**n**	**%**
Correta	20	35,7%	163	63,9%	47	53,4%
Incorreta	36	64,3%	92	36,1%	41	46,6%
Total	56	100%	255	100,0%	88	100%

Com relação aos tipos de erros nas prescrições da quimioprofilaxia, não foram observadas diferenças estatísticas entre as avaliações (Tabela 3). Entretanto, após um ano sem aulas, houve uma tendência de distribuição diferente das avaliações antes das aulas (p = 0,091). Os percentuais de casos incorretos do tipo “não podiam, mas fizeram profilaxia” e “precisavam, mas não fizeram” diminuíram, demonstrando um esclarecimento da sua necessidade. Já os percentuais do tipo “não precisavam, mas fizeram” e “fizeram profilaxia não padronizada” aumentaram, demonstrando uma preocupação com a sua realização, provavelmente influenciada pela conscientização após as aulas e por se tratar de um corpo clínico fechado.

**Tabela 3:** Erros nas prescrições de quimioprofilaxia.

**Profilaxia**	**Antes das aulas de conscientização (maio/2012)**		**Após as aulas de conscientização**		**Após um ano sem conscientização (2015)**	
	**n**	**%**	**n**	**%**	**n**	**%**
Precisava, mas não fez profilaxia	20	55,6%	46	50,0%	16	39,0%
Não precisava, mas fez profilaxia	6	16,7%	22	23,9%	15	36,6%
Fez profilaxia não padronizada	6	16,7%	10	10,9%	8	19,5%
Não podia, mas fez profilaxia	4	11,1%	14	15,2%	2	4,9%
Total	36	100,0%	92	100,0%	41	100,0%

## DISCUSSÃO

O paciente oncológico tem uma predisposição maior para desenvolver TVP. No estudo RIETE, quase metade dos pacientes que desenvolveram TEV durante a internação eram portadores de neoplasia, o que indica ser esta uma população de alto risco de desenvolvimento e recorrência, acarretando custos maiores na gestão do TEV^20^^,^ 21. O risco de morrer depois de um evento trombótico agudo é quatro a oito vezes maior em pacientes com câncer do que em pacientes sem câncer, e o TEV é a segunda causa de óbito em pacientes oncológicos^22^^,^ 23.

Nos estudos de Fuzinatto et al.^6^, Engerhorn et al.^7^, Pereira et al.^9^, Andrade et al.^10^, Rocha et al.^11^ e Franco et al.^24^, identificou-se que a profilaxia do TEV em pacientes internados foi subutilizada, sendo que praticamente a totalidade dos pacientes analisados nos respectivos hospitais tinham alto risco para TEV e que menos da metade deles recebeu profilaxia adequada. Desse modo, medidas profiláticas adequadas não foram utilizadas mesmo em pacientes com fatores de risco potenciais para desenvolvimento de TEV e suas complicações. O mesmo fato ocorreu nesta análise, na qual foi possível verificar que a preocupação com os fenômenos tromboembólicos nos pacientes oncológicos internados por eventos clínicos ou mesmo para fins cirúrgicos foi muito baixa.

O baixo índice de prescrição de profilaxia para o TEV não é um problema somente nacional. O estudo multicêntrico ENDORSE, realizado em 2008 em 32 países com mais de 68 mil pacientes, revelou que mais da metade de todos os pacientes hospitalizados estão em risco de TEV e que os pacientes cirúrgicos parecem estar em maior risco do que os pacientes internados por motivos clínicos. Além disso, apenas metade dos pacientes de risco recebeu algum método recomendado de profilaxia. Verificou-se também que o uso de profilaxia recomendada do TEV foi particularmente insuficiente em pacientes clínicos, sendo que apenas 37% dos pacientes com doença maligna e acidente vascular cerebral isquêmico (dois grupos considerados de mais alto risco para TEV) receberam profilaxia^25^. Outro exemplo foi no serviço de saúde inglês, no qual constatou-se que mais da metade dos pacientes que falecem de EP não recebem tromboprofilaxia, apesar de apresentarem fatores de risco e não apresentarem contraindicação^26^^,^ 27.

A baixa importância dada à profilaxia de TEV pode ser resultado da pouca valorização do TEV como entidade clínica, por ter apresentação clínica inespecífica e dificuldade de diagnóstico objetivo, e estar associado ao temor de sangramentos, principalmente no pós-operatório, e pelo custo financeiro que a quimioprofilaxia pode acarretar. Porém, o seu uso, quando indicado corretamente, apresenta uma relação custo-benefício positiva^26^. A questão financeira, embora relevante na utilização adequada dos recursos, não é superior às necessidades do paciente do ponto de vista ético e científico – o que deve prevalecer sempre, na boa medicina, é o bem-estar do paciente^6^^,^ 10.

A Associação Americana de Oncologia Clínica determina que a maioria dos pacientes hospitalizados com câncer ativo recebam tromboprofilaxia durante toda a internação. Porém, em muitos casos, como nos pacientes submetidos a cirurgias abdominais pélvicas ou de grande porte, considerados de alto risco, a profilaxia deverá ser estendida após a alta do paciente em até quatro semanas^28^. A profilaxia de rotina em pacientes internados para quimioterapia ou para procedimentos menores não deve ser realizada, pois não existem dados suficientes para apoiar tal conduta^29^.

A tromboprofilaxia ainda é um desafio em pacientes oncológicos, pois a incidência de TEV pode variar de 1% em certos tipos de câncer a até 20% ou mais em câncer de pâncreas e gliomas malignos. Por outro lado, os benefícios da quimioprofilaxia têm que ser pesados contra os possíveis riscos, principalmente pela possibilidade de sangramentos^30^.

A aplicação de protocolos na prevenção de TEV^15^^,^ 31 ^,^ 32 para pacientes oncológicos internados necessita de avaliação caso a caso devido aos diversos fatores de risco para TEV envolvidos no tratamento, que inclui quimioterapia, terapia hormonal, radioterapia e cirurgia^19^^-^ 22. Associados a essas dificuldades, também existem os fatores de contraindicação à quimioprofilaxia, frequentes nesse perfil de paciente, os quais ocorreram em 22% dos pacientes avaliados neste estudo, sendo 45% destes devido a plaquetopenia e 42% a sangramento ativo. Tais dificuldades contribuíram para que na avaliação inicial, antes da conscientização, fosse verificado que 64% dos pacientes não receberam a profilaxia correta. Destes, 55% precisavam de profilaxia e não a receberam, o que mostra o desconhecimento sobre o assunto ou a falta de preocupação com a sua importância, apesar de diversos estudos mostrarem a alta prevalência de sua ocorrência em pacientes oncológicos^33^^,^ 34.

No entanto, após o PCE, houve uma diminuição de profilaxias erradas, de 64,3% para 36,1% (p < 0,001), sendo que em 23% dos casos houve prescrição de profilaxia para pacientes que não a precisavam, mostrando que houve uma preocupação maior com a prevenção do TEV. Esse resultado corroborou o estudo prospectivo de Anderson et al.^35^, que documentou um aumento na prescrição de profilaxia de 29% para 52% em pacientes hospitalizados com risco potencial para desenvolver TVP, após a instituição de estratégias educacionais com o propósito de alertar os profissionais para a importância da prevalência do TEV.

No intuito de confirmar o aumento da prescrição de profilaxia, foi verificado também um aumento de sua utilização pela farmácia clínica, principalmente de enoxaparina, até atingir um número três vezes maior de utilizações intra-hospitalares em relação à frequência de utilização antes da semana de conscientização. Quanto à utilização de enoxaparina, foi verificado que o custo direto foi muito parecido com o da HNF, além da vantagem de utilizar de maneira menos frequente os profissionais da saúde, com menos materiais descartáveis e mais conforto ao paciente. Tais vantagens ocorrem pelo fato de ser administrada uma vez ao dia.

Foi possível observar neste estudo que após um ano sem aulas, houve uma tendência de distribuição diferente das avaliações após as aulas (p = 0,091). Os percentuais de casos incorretos do tipo “não podiam, mas fizeram profilaxia” e “precisavam, mas não fizeram” diminuíram, o que demonstra um esclarecimento da correta necessidade da quimioprofilaxia. Já os percentuais do tipo “não precisavam, mas fizeram” e “fizeram profilaxia não padronizada” aumentaram, o que demonstra uma preocupação com a realização da quimioprofilaxia, provavelmente influenciada pelas aulas de conscientização. Isso indica a necessidade de um programa de divulgação e orientação continuada intra-hospitalar, que deve ser associado a outros mecanismos para melhorar a aderência dos médicos à avaliação e prescrição da quimioprofilaxia.

Esclarecimentos sobre a necessidade de quimioprofilaxia, incluindo aulas de orientação, envolvimento de demais profissionais da saúde, como enfermeiros e fisioterapeutas, e criação e implementação de protocolos informatizados, auxiliam na melhoria da prescrição da profilaxia. No entanto, tais situações ainda não foram suficientes para a realização de profilaxia adequada nos pacientes internados. Segundo Rocha et al.^11^ e Maffei et al.^36^, essas medidas são insuficientes para melhorar de forma adequada a prevenção.

Deve-se, além das medidas mencionadas acima, fazer com que os médicos do corpo clínico participem continuamente dos PCEs, fornecendo a eles o conhecimento sobre as estatísticas da doença tromboembólica no hospital onde trabalham e um retorno sobre o seu desempenho e sobre os resultados da profilaxia de seus pacientes^16^^,^ 37. Outra situação que dificulta a prescrição correta de profilaxia é a necessidade de avaliação diária do risco de TEV. No entanto, com a utilização de um programa de alerta eletrônico, é possível aumentar o uso de profilaxia, permitindo identificar e registrar pacientes que inicialmente tiverem um escore de risco de TEV baixo, mas que poderá aumentar durante a hospitalização, reduzindo o risco de TVP ou EP em 90 dias em até 41%^38^^,^ 39. Tais alertas podem otimizar a profilaxia do TEV, ocasionando menor impacto econômico institucional e, o mais importante, redução da morbidade e mortalidade^40^.

A quimioprofilaxia é subutilizada nos pacientes internados, principalmente nos oncológicos, sendo necessária a realização de programas de esclarecimento da importância da realização da prevenção do TEV para que haja uma otimização inicial de sua utilização. Para que ocorra uma melhoria ainda mais acentuada na sua prescrição, recomenda-se formar uma comissão ativa de prevenção do TEV, para que esta inicie um estudo para avaliar a situação atual do hospital e desenvolva estratégias para melhorá-la, envolvendo médicos, enfermeiros, fisioterapeutas, farmácia clínica e setores administrativos. Deverá também manter educação continuada sobre o assunto e utilizar ferramentas de monitoramento, como alertas humanos, na forma de auditorias regulares, e alertas eletrônicos.
