# Supplementary material for: Prevenção de tromboembolismo venoso em hospital com perfil oncológico: como melhorá-la?
Source: J Vasc Bras. 2016 Jul-Sep;15(3):189–96. [Article in Portuguese] doi: 10.1590/1677-5449.003216 (PMC5829755; doi:10.1590/1677-5449.003216)
Supplement: Translation

# Translation: How can prevention of venous thromboembolism be improved in a hospital with an oncological profile?

**Authors:** Fabiano Luiz Erzinger, Marcela Bechara Carneiro

**Affiliations:** 1 Hospital Erasto Gaertner, Serviço de Cirurgia Vascular, Curitiba, PR, Brazil.; 2 Hospital Erasto Gaertner, Setor de Farmácia Hospitalar, Curitiba, PR, Brazil.

**Keywords:** cancer, thromboembolism, prevention

## Abstract

**Background:**

Complications of venous thromboembolism are common among both medical and surgical hospital patients.

**Objective:**

To identify what, if any, pharmacological prophylaxis for venous thromboembolism was given to cancer patients in hospital before and after implementation of a program to raise awareness of its importance.

**Methods:**

This was a cross-sectional study conducted in three phases at distinct times: before a program to raise awareness of the importance of prophylaxis against venous thromboembolism was implemented, during years when the program was being run, and 1 year after the end of the program. For statistical analysis, patients were classified as high risk or no risk and categorized on the basis of erroneous pharmacological prophylaxis, as follows: “needed prophylaxis, but were not given it”; “did not need prophylaxis, but were given it”; “were given nonstandard prophylaxis”; or “should not have been given prophylaxis, but were given it”.

**Results:**

A total of 399 hospital patients were assessed: 56 before the awareness-raising program, 255 during the program and 88 1 year after the program was last run. Before any awareness-raising weeks, just 35.7% of the patients were being given the correct pharmacological prophylaxis; after awareness-raising weeks, the proportion of correct prescriptions increased to 63.9% (p < 0.001). After one year with no awareness-raising efforts, maintenance of pharmacological prophylaxis was no longer as effective, and there was a trend for the proportion of incorrect prophylaxis to increase (p = 0.081).

**Conclusions:**

Pharmacological prophylaxis is given to a very small percentage of patients in hospital and programs are needed to raise awareness of its importance in the prevention of venous thromboembolism and continuous monitoring is needed to facilitate prescriptions.

## INTRODUCTION

The primary cause of avoidable deaths in hospital is pulmonary thromboembolism (PTE), which, in the majority of cases, is a consequence of deep venous thrombosis (DVT). This combination of PTE and DVT is known as venous thromboembolism (VTE) and is a common complication during and after hospital admission, whether admission is for acute medical disease or for surgery.^1^^-^ 3

In addition to the acute risk of mortality, VTE is also associated with long-term risk of development of post thrombotic syndrome and chronic pulmonary hypertension. These complications make a substantial contribution to morbidity, time off work and healthcare management costs.^4^

Approximately 70% to 80% of pulmonary emboli (PE) diagnosed post mortem have no prior diagnosis or clinical suspicion because of their cause and because the complications are often silent. These characteristics mean that prophylaxis should be adopted as a safe and effective measure for patients who have the risk factors for embolism.^5^

Cancer alone is itself a significant risk factor for occurrence of VTE. When cancer patients undergo surgical procedures they are at double the risk of DVT and three times the risk of PTE than patients who do not have cancer,^6^ which is a clear indication of the increased need for attention to providing preventative care for this subset of patients.

Over the last 20 years, studies have been conducted to determine the situation in Brazilian hospitals with relation to VTE prophylaxis and the findings show that among both medical and surgical patients appropriate prophylactic measures are not being taken, even when risk factors for VTE are present.^7^^-^ 11 However, few studies have been conducted with the objective of formulating strategies and providing guidance in the form of hospital-wide thromboprophylaxis programs.^11^^,^ 12

Machado^13^ recommends that a multidisciplinary team should be assembled made up of people who understand the importance of pharmacological prophylaxis, in order to improve the frequency and quality of provision of VTE prophylaxis.^14^^,^ 15 In turn, a study published by Rocha et al.^11^ proposed that a commission should be created in all hospitals with a mission to encourage VTE prophylaxis using educational lectures.

The objective of this study was to evaluate the quality of pharmacological prophylaxis against VTE in medical and surgical patients admitted to an oncological hospital, before and after implementation of a continuing education program designed to raise awareness about the importance and necessity of these procedures.

## METHOD

A cross sectional study involving one-day data collection efforts was conducted at the Erasto Gaertner oncological teaching hospital in three separate phases: before the program to raise the awareness of the importance of VTE prophylaxis in patients in hospital was created, during implementation of the awareness-raising program, and 1 year after the program had been discontinued.

The local institutional research ethics commission gave approval for all phases of the study (certificate number 05040012.9.0000.0098) and all patients studied signed free and informed consent forms.

The awareness-raising program was run in 2012, 2013 and 2014 and comprised 1 week of standardized ongoing education lectures given by specialists in the area of VTE and attended by physicians in the clinical team, residents, nurses, physiotherapists and the clinical pharmacy staff. The lectures covered epidemiological data on prophylaxis for medical and surgical patients, the Brazilian national guidelines on prophylaxis for VTE, the importance of daily patient assessments, and the precautions that must be taken when administering prophylactic medications. In order to help attendees to assimilate the importance of prophylaxis prescription and decision-making, in addition to the lectures the program also involved discussions of clinical cases and distribution of educational material on algorithms for prophylaxis that are contained in the hospital’s electronic patient record and which are different for patients in hospital for general medical conditions than for those admitted for surgery.

The data collected on risk factors for VTE and the pharmacological prophylaxis provided for each patients were those required to fill out the protocols for medical patients and for surgical patients previously published in a supplemental issue of this journal,^16^^-^ 19 and they were acquired by questioning the patients or their legal representatives directly and by consulting their electronic patient records, with the objective of improving the quality of the information collected on the patients’ conditions and their comorbidities. All patients who were in hospital on the day of data collection and gave permission for participation in the study (or whose legal representative gave permission) after were enrolled having read the informed consent form and having been given explanations about the study and about any doubts they raised. Exclusion criteria were: patients under 18 years of age, expectant mothers, patients being treated for VTE, patients who were scheduled for surgery the same day, patients who would be discharged that day, and patients for whom it was not possible to obtain all the information needed to complete the protocol.

Data were collected once before the VTE prophylaxis information lectures were given and between 1 and 3 months after the awareness raising weeks in 2012, 2013 and 2014. In 2015, no VTE awareness-raising activities were conducted and the data collection day was 1 year after the 2014 data were collected. During the study period, the physicians were not informed in advance on which days the patients would be interviewed. For the purposes of statistical analysis, patients were divided into high risk or low risk for VTE. Patients were defined as high risk if they needed prophylaxis or were moderate risk but still needed prophylaxis and patients were defined as low risk if they did not need prophylaxis. Additionally, patients who were managed incorrectly in terms of pharmacological prophylaxis were further stratified according to four possible situations: needed prophylaxis but were not given it; did not need prophylaxis, but were given it; were given nonstandard prophylaxis; or should not have been given prophylaxis, but were given it.

Statistical analysis was conducted to detect associations between prophylaxis classifications during the three study phases (before awareness-raising, after awareness-raising programs, and 1 year after awareness-raising had ceased). Chi-square test results with p values < 0.05 were considered statistically significant. Data were analyzed with IBM SPSS Statistics v.20.0®.

## RESULTS

A total of 399 patients admitted to the Erasto Gaertner hospital were assessed from 2012 to 2015. The sample comprised 226 medical patients and 173 surgical patients (Table 1), the majority of whom were at high risk of developing VTE (66.41%). The subset of surgical patients had a higher percentage of high risk patients (75.14%).

**Table 1:** Hospitalized patients and risk of VTE.

**Assessment phases**	**Risk/patient**	**Treatment**		
		**Medical**	**Surgical**	**Overall**
Before awareness-raising classes (May/2012)	High	19 (59.4%)	22 (91.7%)	41 (73.2%)
	Low	13 (40.6%)	2 (8.3%)	15 (26.8%)
	Total	32	24	56
Soon after awareness-raising classes	High	86 (65.6%)	80 (64.5%)	166 (65.1%)
	Low	45 (34.4%)	44 (35.5%)	89 (34.9%)
	Total	131	124	255
After 1 year without awareness raising (2015)	High	30 (47.6%)	23 (92%)	53 (60.2%)
	Low	33 (52.4%)	2 (8%)	35 (39.8%)
	Total	63	25	88

The most frequent risk factor for VTE was oncological disease (87.78%). Among the patients admitted for medical conditions this was the case in 96.31% and among the surgical patients the rate was 78.37%. In the subset of medical patients, the next most common risk factors were as follows: prolonged immobilization (64.41%), age greater than 55 years (57.66%), and infection (34.35%). Contraindications against pharmacological prophylaxis were present in 90 patients (22.55%), the most common of which were thrombocytopenia below 50,000/mm^3^ (45.71%) and active bleeding (42.85%). These factors were present in approximately 1/4 (26.41%) of the patients at high risk of developing VTE.

A total of 399 patients who were admitted during the study period were assessed, 56 patients before the awareness-raising program was started, 255 patients during the 3 years in which the program was run, and 88 patients 1 year after the last time the program was run.

Overall, the correct pharmacological prophylaxis was prescribed to 213 patients (53.38%). The great majority of the 46.62% of the overall sample who were managed incorrectly in terms of pharmacological prophylaxis, 58 patients (52.68%), were at high risk of VTE, but were not given pharmacological prophylaxis. Low molecular weight heparin was given to 87.2% of the patients, because the difference in cost in relation to the unfractionated heparin was minimal (less than 3% per day), without taking into account the difference in indirect costs and posology.

Before the awareness-raising week, just 35.7% of patients were being given the correct pharmacological prophylaxis. More than half of the 36 patients who were managed incorrectly (55.6%) were high-risk patients, but were not given prophylaxis.

After the awareness-raising week, there was a statistically significant increase in the proportion of correct pharmacological prophylaxis decisions, which rose from 35.7% to 63.9% (p < 0.001), which demonstrates the effectiveness of the program run at the hospital. However, this was still insufficient, since around 1/3 (186) of the patients admitted during this period were managed incorrectly in this respect. On the other hand, there was a non-significant trend to prescribe pharmacological prophylaxis more often, and 23.9% of patients who did not need it because they were low risk were prescribed it regardless, which demonstrates concern with assessing patients’ need for protection against VTE (Figure 1).

**Figure 1:** Pharmacological prophylaxis performance over the course of the study.

Additionally, even after 1 year with no awareness-raising classes (Table 2), the improvement in pharmacological prophylaxis prescription was maintained. The results were not as good as during years in which the week of lectures was held, since there was a trend for incorrect prophylaxis management to increase (p = 0.081), but the number of incorrectly managed patients was lower than before the classes (p < 0.001).

**Table 2:** Maintenance of correct prophylaxis after awareness-raising exercises.

**Prophylaxis**	**Before awareness-raising classes (May/2012)**		**Soon after awareness-raising classes**		**After 1 year without awareness raising (2015)**	
	**n**	**%**	**n**	**%**	**n**	**%**
Correct	20	35.7%	163	63.9%	47	53.4%
Incorrect	36	64.3%	92	36.1%	41	46.6%
Total	56	100%	255	100.0%	88	100%

With regard to the types of pharmacological prophylaxis prescription errors, there were no statistically significant differences between the three periods (Table 3). However, after 1 year without classes, there was a trend towards a different distribution from the distribution of the results for the period before the classes (p = 0.091). The percentages of incorrect cases of the type “should not have been given prophylaxis, but were given it” and the type “needed prophylaxis, but were not given it” reduced, demonstrating greater understanding of the need for prophylaxis. However, the percentages of the type “did not need prophylaxis, but were given it” and “were given nonstandard prophylaxis” both increased, demonstrating greater concern with providing prophylaxis, probably influenced by the greater awareness achieved after the classes and because the hospital is exclusively staffed by a home team.

**Table 3:** Errors in pharmacological prophylaxis prescription decisions.

**Prophylaxis**	**Before awareness-raising classes (May/2012)**		**Soon after awareness-raising classes**		**After 1 year without awareness raising (2015)**	
	**n**	**%**	**n**	**%**	**n**	**%**
Needed prophylaxis, but not given it	20	55.6%	46	50.0%	16	39.0%
Did not need prophylaxis, but given it	6	16.7%	22	23.9%	15	36.6%
Given nonstandard prophylaxis	6	16.7%	10	10.9%	8	19.5%
Should not have been given prophylaxis, but given it	4	11.1%	14	15.2%	2	4.9%
Total	36	100.0%	92	100.0%	41	100.0%

## DISCUSSION

Cancer patients have a greater predisposition to develop DVT. In the RIETE study, almost half of the patients who developed VTE while in hospital had neoplasms, indicating that this is a population at high risk of both development and recurrence, increasing the cost of VTE management.^20^^,^ 21 The risk of death after an acute thrombotic event is four to eight times greater in patients with cancer than in patients without cancer and VTE is the second most common cause of death among cancer patients.^22^^,^ 23

Studies by Fuzinatto et al.,^6^ Engerhorn et al.,^7^ Pereira et al.,^9^ Andrade et al.,^10^ Rocha et al.^11^ and Franco et al.,^24^ all observed that prophylaxis against VTE was under-utilized in patients in hospital, and practically all of the patients analyzed at the hospitals involved had high VTE risk, but less than half of them were given the appropriate prophylaxis. As such, the correct prophylactic measures were not taken, even in patients with potential risk factors for development of VTE and its complications. The same was observed in this analysis, in which it was observed that the level of concern for thromboembolic phenomena in cancer patients admitted to hospital for general medical events or for surgery was very low.

The problem of low rates of prescriptions of prophylaxis against VTE is not restricted to Brazil. The ENDORSE multicenter study was conducted in 32 different countries in 2008 with more than 68 thousand patients, finding that more than half of all hospitalized patients were at risk of VTE and that surgical patients appear to be at greater risk than patients admitted for medical reasons. Furthermore, just half of at-risk patients were given a recommended prophylaxis method. It was also observed that use of the recommended prophylaxis against VTE was particularly deficient in medical patients, since just 37% of patients with malignant disease and ischemic stroke (two groups considered at greatest risk of VTE) were given prophylaxis.^25^ Another example was in the English national health service, where it was found that more than half of patients who died from PE had not been given thromboprophylaxis, despite having risk factors and not having contraindications.^26^^,^ 27

It is possible that the low importance attributed to prophylaxis against VTE is itself a result of undervaluing VTE as a clinical entity, because it has nonspecific clinical presentation and is difficult to diagnose objectively, and may also be linked to fears of bleeding, particularly during the postoperative period, and to the increased financial burden that pharmacological prophylaxis can impose. However, when correctly indicated, prophylaxis can have a positive cost-benefit profile.^26^ While the financial element is relevant to making the best possible use of the resources available, it does not take precedence over the needs of patients from either an ethical or a scientific point of view – since in good medical practice, patient wellbeing must always come first.^6^^,^ 10

The American Society of Clinical Oncology recommends that the majority of hospitalized patients with active cancer should be given thromboprophylaxis throughout their hospital stay. Furthermore, in many cases, such as in patients who undergo pelvic, abdominal or major surgery, who are considered high risk, prophylaxis should be continued for up to 4 weeks after patient discharge.^28^ Routine prophylaxis should not be given to patients admitted for chemotherapy or for minor procedures, because there are not enough data to support this approach.^29^

Thromboprophylaxis remains a challenge in cancer patients, because the incidence of VTE can vary from 1% in certain types of cancer up to 20% or more in pancreatic cancer and malignant gliomas. On the other hand, the benefits of pharmacological prophylaxis must be weighed against the possible risks, primarily the possibility of bleeding.^30^

Application of protocols for prevention of VTE^15^^,^ 31 ^,^ 32 in cancer patients in hospital demands case-by-case assessment because of the large number of risk factors for VTE involved in cancer treatment, including chemotherapy, hormone therapy, radiotherapy and surgery.^19^^-^ 22 Compounding these difficulties, there are also many factors that contraindicate pharmacological prophylaxis, which are frequently present in this patient profile. In the present study, 22% of the patients assessed had contraindications, 45% due to thrombocytopenia and 42% because of active bleeding. These difficulties contributed to the finding that at the first assessment point, before the awareness-raising program, 64% of the patients were not being prescribed the correct prophylaxis. Of these, 55% needed prophylaxis, but were not given it, which illustrates either a lack of knowledge of the subject or insufficient concern for its importance, despite the fact that many different studies have shown that VTE prevalence is elevated in cancer patients.^33^^,^ 34

However, after the awareness-raising program, the proportion of patients subject to incorrect prophylaxis management reduced from 64.3% to 36.1% (p < 0.001), and in 23% of cases, prophylaxis was prescribed for patients who did not need it, showing that concern for VTE prevention had increased. This finding supports the results of a prospective study conducted by Anderson et al.,^35^ who found that after implementation of educational strategies intended to alert health professionals to the importance of VTE prevalence, the proportion of prophylaxis prescription increased from 29% to 52% in hospitalized patients with a potential risk of developing DVT.

In order to confirm the increase in prophylaxis prescription, data were also collected from the clinical pharmacy, showing that use had increased, particularly of enoxaparin, which reached a rate of intrahospital use three times greater than before the awareness-raising week. With regard to use of enoxaparin, it was found that the direct cost was very similar to the cost of unfractionated heparin, in addition to offering the advantage of less frequent use of health professionals’ time, with less disposable materials used, and increased comfort for patients. These advantages are because it is administered once a day.

It was also observed in the present study that, after 1 year without classes, there was a trend towards a different distribution compared to assessments conducted soon after the classes (p = 0.091). The percentages of cases of incorrect management of the types “should not have been given prophylaxis, but were given it” and “needed prophylaxis, but were not given it” reduced, which shows that awareness of the true need for pharmacological prophylaxis had improved. The percentages of incorrect decisions of the types “did not need prophylaxis, but were given it” and “were given nonstandard prophylaxis” both increased, which illustrates greater concern with administration of pharmacological prophylaxis, probably influenced by the awareness-raising classes. This illustrates the need for a continuous program to publicize and provide guidance, which should be combined with other mechanisms for improving physicians’ compliance with assessment and prescription of pharmacological prophylaxis.

Efforts to raise awareness of the need for pharmacological prophylaxis, including educational classes, involving other classes of health professional, such as nurses and physiotherapists, and creation and implementation of computerized protocols, all help to improve the quality of prophylaxis prescription decisions. However, these measures were still insufficient to achieve high levels of correct prophylaxis for hospitalized patients. According to Rocha et al.^11^ and Maffei et al.,^36^ these measures are not enough to improve prevention sufficiently.

In addition to the measures listed above, it is also necessary to ensure that the physicians who make up the clinical team continuously take part in awareness-raising exercises providing them with information on the statistical profile of thromboembolic disease in the hospital in which they work and giving them feedback on their performance and the results of the prophylaxis prescribed to their patients.^16^^,^ 37 Another element that makes correct prescription of prophylaxis more difficult is the need for daily assessments of the risk of VTE. However, implementation of an electronic alert program is capable of increasing prophylaxis utilization, making it possible to identify and log patients who initially have low risk of VTE scores, but who may be subject to increasing risk as their hospital stay progresses, thereby reducing the risk of DVT or PE at 90 days by as much as 41%.^38^^,^ 39 Such alerts can lead to optimization of prophylaxis against VTE, resulting in a lower institutional economic impact and, most importantly, reductions in morbidity and mortality.^40^

Pharmacological prophylaxis is under-utilized in patients in hospital, particularly cancer patients, and it is necessary to conduct programs to raise awareness of the importance of measures for prevention of VTE in order to achieve initial optimization of utilization. In order to achieve more accentuated improvements in prophylaxis prescription, it is recommended that an active commission for VTE prevention should be set up and given responsibility for conducting studies to evaluate the current situation at the hospital and for developing strategies to improve it that involve physicians, nurses, physiotherapists, the clinical pharmacy, and administrative departments. Ongoing education on the subject should also be provided and monitoring tools should be implemented, such as human alerts, in the form of regular audits, and electronic alerts.
